# Ending publication bias: A values-based approach to surface null and negative results

**DOI:** 10.1371/journal.pbio.3003368

**Published:** 2025-09-24

**Authors:** Stephen Curry, Eunice Mercado-Lara, Virginia Arechavala-Gomeza, C. Glenn Begley, Christophe Bernard, René Bernard, Stefano Bertuzzi, Needhi Bhalla, Dawn Bowers, Samuel Brod, Christopher Chambers, Michael R. Dougherty, Yensi Flores Bueso, Stefânia Forner, Alexandra L. J. Freeman, Magali Haas, Darla P. Henderson, Kanika Khanna, Rebecca Lawrence, Kif Liakath-Ali, Christine Liu, Neil Malhotra, José G. Merino, Edward Miguel, Rachel Miles, Mary Munson, Shinichi Nakagawa, Robert Nobles, Joy Owango, Michel Tuan Pham, Gina Poe, Alexandra N. Ramirez, Sarvenaz Sarabipour, Jill L. Silverman, Laura N. Smith, P. Sriramarao, Paul W. Sternberg, Geeta K. Swamy, Malú Gámez Tansey, Gonzalo E. Torres, Erick H. Turner, Lauren von Klinggraeff, Frances Weis-Garcia

**Affiliations:** 1 Department of Life Sciences, Imperial College London, London, United Kingdom; 2 Research on Research Institute, London, United Kingdom; 3 Open Research Community Accelerator, San Francisco, California, United States of America; 4 Nucleic Acid Therapeutics for Rare Diseases, Biobizkaia Health Research Institute, Barakaldo, Spain; 5 Ikerbasque, Basque Foundation for Science, Bilbao, Spain; 6 Begley Biotech Consulting, Melbourne, Victoria, Australia; 7 Aix Marseille Université, INSERM, Institut de Neurosciences des Systèmes, Marseille, France; 8 Excellenzcluster NeuroCure, Charité – Universitätsmedizin Berlin, Berlin, Germany; 9 American Society for Microbiology, Washington, District of Columbia, United States of America; 10 Department of Molecular, Cell and Developmental Biology, University of California, Santa Cruz, Santa Cruz, California, United States of America; 11 Department of Clinical and Health Psychology, University of Florida, Gainesville, Florida, United States of America; 12 BioMed Central, London, United Kingdom; 13 School of Psychology, Cardiff University, Cardiff, United Kingdom; 14 Department of Psychology, University of Maryland, College Park, Maryland, United States of America; 15 Cancer Research @UCC, University College Cork, Cork, Republic of Ireland; 16 Institute for Protein Design, University of Washington, Seattle, Washington, United States of America; 17 Global Young Academy, Halle, Germany; 18 Alzheimer’s Association, Chicago, Illinois, United States of America; 19 Octopus Publishing CIC, Abingdon, United Kingdom; 20 Cohen Veterans Bioscience, New York, New York, United States of America; 21 Federation of American Societies for Experimental Biology, Rockville, Maryland, United States of America; 22 Gladstone Institute of Virology, University of California, San Francisco, San Francisco, California, United States of America; 23 F1000 Research Ltd, Taylor & Francis Group, London, United Kingdom; 24 School of Biological Sciences, University of Southampton, Southampton, United Kingdom; 25 Department of Psychiatry, University of California, San Francisco, San Francisco, California, United States of America; 26 Graduate School of Business, Stanford University, Stanford, California, United States of America; 27 Department of Neurology, Georgetown University Medical Center, Washington, District of Columbia, United States of America; 28 Department of Economics and Center for Effective Global Action, University of California, Berkeley, Berkeley, California, United States of America; 29 University Libraries, Virginia Tech, Blacksburg, Virginia, United States of America; 30 Department of Biochemistry and Molecular Biotechnology, University of Massachusetts Chan Medical School, Worcester, Massachusetts, United States of America; 31 Department of Biological Sciences, University of Alberta, Edmonton, Alberta, Canada; 32 Office of Research Administration, Emory University, Atlanta, GeorgiaUnited States of America; 33 Training Centre in Communication, University of Nairobi, Nairobi, Kenya; 34 Columbia Business School, Columbia University, New York, New York, United States of America; 35 Integrative Biology and Physiology Department, University of California, Los Angeles, Los Angeles, California, United States of America; 36 Neuroscience Program, Icahn School of Medicine at Mount Sinai, New York, New York, United States of America; 37 Department of Cell Biology, University of Connecticut School of Medicine, Farmington, Connecticut, United States of America; 38 Department of Psychiatry and Behavioral Sciences, MIND Institute, School of Medicine, University of California, Davis, Sacramento, California, United States of America; 39 Department of Neuroscience and Experimental Therapeutics, Texas A&M University College of Medicine, Bryan, Texas, United States of America; 40 Office of the Vice President for Research and Innovation, Virginia Commonwealth University, Richmond, Virginia, United States of America; 41 Division of Biology and Biological Engineering, California Institute of Technology, Pasadena, California, United States of America; 42 Department of Obstetrics and Gynecology, Duke University, Durham, North Carolina, United States of America; 43 McKnight Brain Institute, Department of Neuroscience, University of Florida Health System, Gainesville, Florida, United States of America; 44 Department of Molecular Pharmacology and Neuroscience, Loyola University Chicago Stritch School of Medicine, Chicago, Illinois, United States of America; 45 Department of Psychiatry, Oregon Health & Science University, Portland, Oregon, United States of America; 46 Department of Community and Behavioral Health Sciences, Institute of Public and Preventive Health, School of Public Health, Augusta University, Augusta, GeorgiaUnited States of America; 47 Antibody and Bioresource Core Facility, Memorial Sloan Kettering Cancer Center, New York, New York, United States of America

## Abstract

Sharing knowledge is a basic tenet of the scientific community, yet publication bias arising from the reluctance or inability to publish negative or null results remains a long-standing and deep-seated problem, albeit one that varies in severity between disciplines and study types. Recognizing that previous endeavors to address the issue have been fragmentary and largely unsuccessful, this Consensus View proposes concrete and concerted measures that major stakeholders can take to create and incentivize new pathways for publishing negative results. Funders, research institutions, publishers, learned societies, and the research community all have a role in making this an achievable norm that will buttress public trust in science.

## Abstract 2

Sharing knowledge is a fundamental principle within the scientific community, yet null and negative results are still being underreported. This Consensus View discusses the problem of such publication bias and presents a roadmap to a solution that has a role for everyone in the scientific community in promoting knowledge sharing, research transparency and rigor.

## Introduction

Scientific progress relies on the dissemination of knowledge so that others can build upon it. Yet not all scientific knowledge is disseminated [1]. Studies with ‘nonsignificant’ or ‘unexciting’ results often remain unpublished, a phenomenon known as ‘the file drawer problem’ or publication bias [2]. This bias persists despite decades of warnings against its consequences [3–8]. By its very nature, the extent of publication bias is difficult to ascertain, but the available data clearly indicate that null findings are underreported. For instance, according to recent studies, fewer than 2 in 100 articles on prognostic markers or animal models of stroke report null findings [9,10], a result that is unlikely to arise from a system that faithfully reports the outcome of experiments regardless of statistical significance. More concretely, published meta-analyses frequently show evidence of publication bias [11], and the introduction of the ‘registered report’ format, in which journals commit to publication before experimental outcomes are known, substantially increased the proportion of null fundings in some psychology journals [12]. Nevertheless, publication bias remains the subject of ongoing debate. In a wide-ranging critique of the so-called ‘crisis narrative’ that has emerged in recent years, Fanelli [13] recognizes the problem but cautions against universalizing it, citing the lack of evidence across many fields. Silvertown and Conway [14] argue that the impact of publication bias may have been overstated due to underestimates of the tendency for false positives to eventually succumb to further scrutiny.

The extent of publication bias does seem to vary across disciplines. For example, surveys of meta-analyses suggest that publication bias is greater in some social science disciplines than it is in biomedical or physical sciences [11,15]. This variation can arise owing to any number of factors, ranging from choice of research methodology (e.g., observational studies, or research that is more descriptive, qualitative, or theoretical) to distinctive disciplinary norms or cultures. Importantly, both the magnitude and consequences of under-reporting of null findings also vary across disciplines. In biomedicine and clinical research, unreported null results can lead to patient-care risks, whereas in fields like economics or ecology, the societal impact of underreported null findings might be less obvious than the impact it has on research efficiency and advancing knowledge [16].

Notwithstanding the modulating effects of methodological and disciplinary variations, the consequences of publication bias can be severe. Unpublished null studies waste resources, slow the pace of science, and impede career advancement. Unwitting researchers are likely to expend time and money conducting similar experiments, not realizing that prior work has yielded null results: time and money that could have been spent pursuing new and more promising ideas. The failure to publish null findings also distorts the literature, resulting in exaggerated effect sizes [17–20], biased meta-analyses [11], inaccurate clinical trial results, flawed policy interventions [21–23], and the acceptance of false claims if incorrect ideas are not challenged [20,24]. Moreover, the rise of artificial intelligence (AI) models trained on incomplete data can magnify this problem, potentially contributing to misinformation [25] and undermining public trust in science.

Publication bias is deeply rooted in scientific culture. Null studies are often perceived as less valuable, leading to harsher peer reviews [26–28] and lower citation rates [29,30]. Null studies are also commonly supposed to be unlikely to be accepted by higher-impact journals [31], further limiting their visibility and reinforcing biases against reporting such findings. Tenure and promotion systems frequently prioritize journal impact factors over methodological rigor [32,33], further discouraging researchers from sharing null results. Additional discouragement arises because reviewers of funding applications may focus on the perceived prestige of the publication outlet rather than the content of investigators’ prior work [34]. As a result, many investigators are reluctant to publish null studies despite the time and effort spent conducting experiments and other empirical studies [35–37]. Even apart from external drivers, investigators may, for good reason, lose confidence in a line of inquiry without having validated or controlled their findings to the rigorous degree that might be applied to a positive result, and therefore be less likely to write them up for publication. There are many ways for an experiment to go awry, and the constant pressure for researchers to focus on what they perceive to be their most productive lines of inquiry may lead them to set aside lines of research that have not yielded positive results.

Journal practices can also exacerbate publication bias. A recent analysis (https://go.nih.gov/looOO3o) by the US National Institute of Neurological Disorders and Stroke (NINDS) found that 180 out of 215 neuroscience journals do not explicitly welcome null studies, while only 14 appeared to accept null studies without additional conditions (e.g., a higher burden of evidence than required for positive studies). Although newer scientific dissemination mechanisms such as study preregistration and Registered Reports [38–41] have shown promise in increasing the publication of null results [12,42], they are not universally applicable [43–47], despite recent efforts to extend their use to exploratory and observational studies [48,49]. Other more recent formats, such as micropublications [50] and modular publications [51], also offer promising avenues for sharing null studies, as do F1000 open research platforms and preprint and data repository platforms such as bioRxiv, arXiv, OSFPreprints, Zenodo, Figshare, and Dryad (bioRxiv even has a dedicated ‘Contradictory Results’ section), all of which can offer more frictionless avenues to dissemination than traditional journals [52,53]. However, these platforms still underrepresent null findings (https://go.nih.gov/looOO3o).

Given the many and various factors at play in sustaining publication bias, what scope is there to prevent it from happening? In this Consensus View, we present a framework for practical action that seeks to enlist contributions from all the relevant stakeholders within the research ecosystem. Although our perspective is primarily biomedical, we believe there is a fresh opportunity for a wider discussion of the problem of publication bias across all disciplines and the construction of proportionate and effective measures to address it.

## Methodology

This ideas and reflections presented here were generated in discussions by participants of the Novel Approaches to Preventing Publication Bias Workshop (https://go.nih.gov/Y6iYPxU), which was run by the NINDS Office of Research Quality in May 2024 in Bethesda, Maryland, USA. This meeting aimed to assess progress and identify remaining barriers to disseminating null studies across the research ecosystem.

Around 50 stakeholders were invited to ensure broad representation by sector (researchers and research institutions; traditional and nontraditional publishers; journal editors; funders; nonprofit organizations; industry; scientific societies), career stage (senior and early career researchers), and geography (United States of America, United Kingdom, Ireland, Germany, Spain, France, Mexico, Australia, plus virtual participation from Kenya). Although most attendees were drawn from biomedical fields, experts in psychology, economics, business, ecology, and public health were included to capture cross-disciplinary perspectives.

The agenda combined plenary presentations with five sector-focused breakout sessions (researchers, research institutions, traditional and nontraditional publishers, scientific societies and nonprofit organizations, and funders and policymakers), each facilitated by a domain expert and an early career participant. Sessions were followed by a closing plenary in which all attendees synthesized key insights.

Facilitators recorded breakout session notes using a shared template. These notes were consolidated in the closing plenary, then thematically coded by two independent analysts. Emergent themes were organized into the framework presented here, ensuring that domain-specific nuances and overarching patterns informed the final recommendations.

## Values-based approach to system change

**Fig 1:**
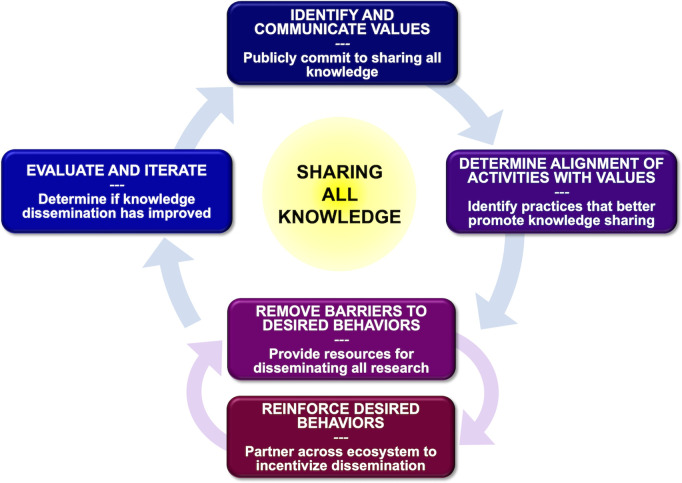
Values-based approach to reducing publication bias. Conceptual diagram illustrating the cyclic and iterative nature of the steps involved in seeking to end publication bias.

To address the causes of publication bias, scholars from diverse disciplinary and geographical backgrounds gathered at a NINDS-hosted meeting in May 2024. They agreed that while scientists should be free to choose which questions to pursue, whatever the results of their research, the current scientific culture clearly incentivizes the production of ‘significant’ or positive results over methodological rigor [54–57]. To transform this culture, we argue that there is a need to shift away from valuing only positive or ‘exciting’ results towards prioritizing the importance of the research question and the quality of the research process, regardless of outcome [55,58,59]. We therefore propose a values-based approach [60–66] to reforming policies, activities, and incentives to reduce or eliminate the occurrence of publication bias (Fig 1). At its core, we believe this means removing barriers to sharing all knowledge (regardless of statistical significance or perceived impact) and enabling, incentivizing and centering the key academic values of transparency, accessibility, and openness.

Drawing principally on experience from biomedical and related science, technology, engineering and mathematics domains, but with relevance to social science, the humanities, and beyond, we therefore call on all sectors of the scientific enterprise to commit openly to the dissemination of all knowledge and to enact specific, practical changes to accomplish this goal within their respective disciplines. We propose a framework for action that includes improved incentive structures to reward transparency and rigor, the creation of simpler mechanisms for reporting null results, and collaboration among sectors of the scientific community to achieve this goal.

Sustaining these changes will require reinforcement from a wide range of actors in the funding, delivery, and dissemination of research. Below, we outline concrete steps for different stakeholders to reduce publication bias.

### Openly commit to the value of disseminating all knowledge

As a first step, we ask all scientific entities (including institutions, departments, core facilities, libraries, ethics committees, scientific societies, funders, journals, laboratory groups, and individual scientists) to reflect on the value of sharing and disseminating all knowledge and to hold internal conversations on why this is crucial for their missions. This is essential to winning internal buy-in from across organizations so that they are positioned to make credible and achievable commitments within their respective domains to sharing all knowledge and eliminating publication bias.

### Identify which practices align with sharing all knowledge

Funders, research-performing organizations, and publishers should assess whether current practices facilitate or hinder transparent research processes. For example, do current practices of researcher assessment (e.g., graduation/degree requirements, promotion, and tenure policies) encourage dissemination of all high-quality research, including null results, or do they focus only on the most ‘exciting’ results (e.g., by heavily weighing bibliometrics or media coverage, which are dubious measures of research quality [67–70])? Does manuscript peer review give due weight to the questions being addressed and the methodological quality of the experiments when determining scientific merit and impact? Does grant review focus on the methodological rigor of investigators’ prior work rather than on the journals in which their papers were published? In many current research settings, the answer is no [55,71]. Explicitly outlining which practices should be modified enables a systematic approach to conceptualizing and implementing these changes, such as through new resources or incentives. Sharing research findings publicly should be a default part of the research workflow rather than an optional step that depends on study outcomes. Commitments to sharing all knowledge should be accompanied by a clear plan of action so that organizations can publicly be held accountable. Table 1 illustrates specific actions for different entities to consider.

**Table 1:** High-priority interventions that promote the dissemination of all knowledge, including null studies, across research fields.

Entity	High-priority activities that align with sharing all knowledge
*Funders*	• Require reporting of all outcomes from previously funded proposals (including work packages not undertaken and null studies) in curricula vitae/biosketches and progress reports, and take this into account during funding considerations. • Support existing platforms and pilots for simplifying and facilitating the default dissemination of all rigorous studies, such as free, funder-backed online venues for disseminating short, quality-controlled null studies linked to their underlying protocols and data. • Modify grant review criteria and train peer reviewers to focus more heavily on the investigator’s history of sharing all knowledge and the methodological rigor of the prior work supporting the research question and of the proposed work (and less heavily on exciting but potentially misleading preliminary outcomes of experiments).
*Research institutions and their sub-entities*	• Reform researcher assessment across all career stages (e.g., training, hiring, promotion and tenure) by enlarging definitions of research ‘excellence’ and ‘impact’ to include ‘pursuing important research questions and performing sound research that is transparently reported’, rather than relying primarily on bibliometric indicators, and by ensuring PhD graduation requirements include reporting of all rigorously performed studies in the dissertation or another public venue, without expectation for positive results. • Provide training to researchers at all career stages on the importance and process of sharing all knowledge, including null studies, within undergraduate and graduate curricula and other professional development programs • Provide infrastructure and logistical support for sharing a variety of research outputs
*Traditional publishers*	• Publicly welcome and actively promote the submission and publication of methodologically sound null studies and Registered Reports • Track submission of null studies to monitor progress • Provide guidance to peer reviewers and editors on how to assess methodology while remaining neutral to the direction (or statistical significance) of outcomes
*Newer research dissemination venues*	• Build upon existing platforms that remove barriers to submission of methodologically sound null findings by including: free submission and publication; simple formats that do not require complete ‘stories’; the capacity to describe full methodology, such as linking to published protocols; pre- or post-publication peer-review of methods; and interconnected data repositories to facilitate data citation.
*Societies & organizations*	• Raise awareness of the importance of null studies, for example, through: hosting discussions of the extent and impacts of publication bias within their disciplines; encouraging submission of null studies to associated journals; developing best practice toolkits and educational workshops; creating early career ambassador programs to advocate for change; featuring null studies at conferences; and providing travel awards to individuals who have performed high-quality studies that happened to produce null results.
*Researchers of all career stages*	• Be a role model for other researchers (including mentees) by sharing null studies publicly. • Advocate for change via roles as colleagues, mentors, peer reviewers, journal editors, institutional administrators, or scientific society officers, and by creating or joining networks of other researchers to share best practices for effecting change.

While the ideas in Table 1 represent possible starting points for different stakeholders, we do not wish to be overly prescriptive in how to effect change. The most effective solutions are likely to be tailored to disciplinary norms and practices and will therefore require ground-level input, but we would nevertheless highlight some ideas for practical implementation by different stakeholders. Funders, for example, could pilot a ‘null-results summary’ program that requires a one-page report of null findings linked to project registries that are accessible to reviewers of subsequent funding applications; such reports could also usefully include mention of work packages or proposed research questions that were not pursued (e.g., because of unanticipated developments or shifts in priorities). Institutions could further support this effort by hosting discipline-specific ‘null-data clinics’, periodic forums where researchers present null results alongside methodological lessons learned. Publishers could place a more explicit emphasis on the review of methodological rigor when handling manuscripts and highlight high-quality papers reporting null findings to encourage submissions. They could also monitor acceptance rates of manuscripts containing positive or negative results to iteratively refine their guidelines for authors and reviewers.

### Enable and reinforce change

Given their influence in the system, research funders (public and private) are uniquely positioned to lead the implementation of policies that incentivize research transparency. Funder policies that are adequately enforced provide strong but proportionate incentives to change researcher reporting behavior and institutional researcher assessment practices. Funders also have the resources to spearhead the development of new infrastructure for easy dissemination outside of traditional publishing venues, for example, by supporting platforms that enable simple and widespread sharing of concise reports of null findings or unfinished projects.

Academic institutions will no doubt want to ensure their researchers are responding to funder incentives, but can synergize in other ways with efforts to address publication bias. For example, when assessing researchers, they should prioritize methodological quality in evaluating experimental outcomes. This could encourage a shift away from reliance on publication prestige and support outlets that emphasize methodological quality and openness. Enhancing education and awareness about effective knowledge sharing will further support these changes.

More ambitious actions, such as creating new tools or platforms for publication and review of null results, will likely require careful staged implementation, piloting, and engagement with different stakeholders (e.g., funders, publishers, researchers, and reviewers). Only with proper incentives and well-designed processes will barriers to the publication of null results be overcome.

### Evaluate and iteratively improve interventions over time

As with any effort to enact genuine reform, a crucial step will be to document, evaluate, and publicly share whether modifications to processes, policies, and practices increase research transparency. At the same time, reformers should be mindful to avoid any negative unintended consequences (e.g., inequity, inappropriate gaming of the system, or undue burden). There are a number of formal evaluation frameworks that may be helpful for guiding the change process [72–74]. These emphasize the importance of a systems-level perspective, collaborating with stakeholders to develop and contextualize the framework, and iterative incorporation of new insights to refine the intervention. Openness to critical evaluation throughout cycles of reform (Fig 1) will be essential if we are to make real progress in addressing publication bias.

## Conclusions

While sharing knowledge is a fundamental principle within the scientific community, publication bias has remained a tough nut to crack and requires renewed attention from the broader research community as part of wider debates about the health of the research and scholarly enterprise. Addressing the challenge of publication bias will not just enhance the progress of research, but also buttress the social contract that publicly funded research relies on for continued support. Our roadmap has a role for all stakeholders (funders, institutions, publishers, and researchers) in promoting knowledge sharing, research transparency, and rigor, but change will only happen if we are all willing to play our part.
